# The Multistream Self: Biophysical, Mental, Social, and Existential

**DOI:** 10.1100/tsw.2008.19

**Published:** 2008-03-25

**Authors:** Vinod D. Deshmukh

**Affiliations:** 3600 Rustic Lane, Jacksonville, Florida 32217, USA

**Keywords:** self, existential, ego, consciousness, self-actualization, being, presence, synergetics, coordination dynamics, well-being, mind, personhood

## Abstract

Self is difficult to define because of its multiple, constitutive streams of functional existence. A more comprehensive and expanded definition of self is proposed. The standard bio-psycho-social model of psyche is expanded to biophysical-mental-social and existential self. The total human experience is better understood and explained by adding the existential component. Existential refers to lived human experience, which is firmly rooted in reality. Existential living is the capacity to live fully in the present, and respond freely and flexibly to new experience without fear. Four common fears of isolation, insecurity, insignificance, and death can be overcome by developing a lifestyle of whole-hearted engagement in the present reality, creative problem solving, self-actualization, and altruism. Such integrative living creates a sense of presence with self-awareness, understanding, and existential well-being. Well-being is defined as a life of happiness, contentment, low distress, and good health with positive outlook. Self is a complex, integrative process of living organisms. It organizes, coordinates, and integrates energy-information within and around itself, spontaneously, unconsciously, and consciously. Self-process is understood in terms of synergetics, coordination dynamics, and energy-information–directed self-organization. It is dynamic, composite, ever renewing, and enduring. It can be convergent or divergent, and can function as the source or target of its own behavior-mentation. The experience of self is continuously generated by spontaneous activation of neural networks in the cerebral neocortex by the brainstem-diencephalic arousal system. The multiple constitutive behavioral-mental streams develop concurrently into a unique experience of self, specific for a person at his/her developmental stage. The chronological neuro-behavioral-mental development of self is described in detail from embryonic stage to old age. Self can be behaviorally-mentally oriented and realized in three complimentary modes of being: egocentric, allocentric, and ecosystemic or existential. The existential mode is both immanent and transcendent, and can be self-actualized, resulting in a healthy, creative, conflict-free, and meaningful life.

## INTRODUCTION

The human sense of self is hard to define since the word has so many connotations. The *Dictionary of Psychology*[1] defines the word "self" as the totality of the individual, consisting of all characteristic attributes, conscious and unconscious, mental and physical. It refers to personal identity, being, and experience. A person can refer to self either as the source of its agency for regulation of behavior, or as the target of perception-cognition-action. It is the essence of individuality – being, proprium, or ipseity. The present or minimal self is the life-in-the-moment or the immediate experience of one's person, unextended in time. Narrative, autobiographical, or longitudinal self is based on autobiographical memories and projected future.

According to the psychologist William James, nominative self is the self as knower of the self, whereas, empirical self is the self so known. The empirical self further consists of the material self, the social self, and the spiritual self, which is closest to one's core subjective experience of oneself. Farah and Heberlein[2] tried to define "personhood" in neuropsychological terms. They proposed "the concept does not correspond to any real category of objects in the world. Rather, it is the product of an evolved brain system that develops innately and projects itself automatically and irrepressibly onto the world whenever triggered by stimulus features such as a human-like face, body, or contingent patterns of behavior."

In this article, I am proposing to extend the standard bio-psycho-social model of mind and self to a more comprehensive concept of bio-psycho-social-existential self and mind. By adding the existential component to the self-concept and mind, the total human experience is better understood and explained. The word "existential" refers to the concrete lived experience of human beings, firmly rooted in the real world. Existential living is the capacity to live fully in the present, and respond freely and flexibly to new experience without fear. Such integrative living would create an experience of presence[3] with an awareness and understanding of one's current existence, and a life of self-actualization and an existential well-being. Well-being is defined as a state of happiness, contentment, low levels of distress, overall good physical and mental health, and a healthy outlook with good quality of living.

Maslow defined self-actualization as "an episode in which powers of the person come together in an efficient and enjoyable way and in which he was more integrated and open to experience, more spontaneous and expressive, fully functioning, more creative, humorous, more ego-transcending, and more independent of his/her lower needs. He becomes more truly himself, more perfectly actualizing his/her potentialities, closer to the core of his/her being, more fully human"[4]. The self-actualized individuals learn to overcome the four existential fears, such as one's nonexistence or death, lack of external support and being on oneself with a need for free choice with responsibility, self-isolation from all creatures in the world, and lack of meaning and hope in life. Yalom[5] suggested whole-hearted engagement or immersion in current life, creativity, self-actualization, and altruism as possible solutions to these ultimate existential fears.

The wisdom of self-integrity is beautifully expressed by Erikson (1902-1994). Meissner comments**:** "Integrity marks the culmination of development of the personality in Erikson's schema. It means acceptance of oneself and all aspects of life and integration of these elements into a stable pattern of living. It implies the experience of and adjustment to the trials and troubles of life as well as to its rewards and joys. Consequently, existence holds no fear; the ego has resigned itself to acceptance of life itself and to acceptance of the end of that life in death. Integrity thus represents the fully developed personality in its most mature self-realization"[6]. "Integrity is acceptance of one's one and only life cycle and of the people who have become significant to it as something that had to be and that, by necessity, permitted of no substitutions."[...] "The individual relinquishes the wish that important people in his/her life had been different and is able to love in a more meaningful way - one that reflects an acceptance of responsibility for one's own life. The individual in possession of the virtue of *wisdom* and a sense of integrity has room to tolerate the proximity of death and achieve what Erikson termed a "detached yet active concern."[...] "The style of integrity developed by his/her culture or civilization thus becomes the 'patrimony' of his/her soul...In such final consolidation, death loses its sting"[7].

I am also using the word “self” from the point of view of new biophysics based on synergetics[8], dynamic patterns[9], coordination dynamics, energy-information–directed self-organization, and holistic-biological perspective of neuroscience[10]. “Synergetics is the cooperation of individual parts of a system that produces (new) macroscopic spatial, temporal and functional structures. It deals with deterministic as well as stochastic processes.” Coordination dynamics is “a set of context-dependent laws or rules that describe, explain, and predict how patterns of coordination form, adapt, persist, and change in natural systems”[11].

In a natural system, like a school of fish, there is an *upward causation* wherein individual fish or part interact and coordinate with each other, and create a new, coordinative macrostructure, i.e., the school of fish, which is a whole with its own new properties and rules of behavior. The school of fish – the whole, in turn, influences the behavior of all fish – a *downward causation*. Two of these effects together constitute *circular causality* in the dynamic natural systems. These concepts are applicable to many complex, natural systems including living organisms and their societies, brain activity, animal behavior, cognition, and mentation.

## ORGANIZATION OF SELF: ITS MULTISTREAM SYNERGIC PATTERN

Self is a complex, integrative process of a living organism. The living organism is a functioning, behaving unit of life. The living organism organizes, coordinates, and integrates information energy within and around itself, spontaneously, unconsciously, and consciously. West[12] summarized the importance of neural integration: “How can diverse neuronal mechanisms provide coherent subjective experiences and volition? The answer is *integration.* Every living creature - beginning with the most primitive – is fully integrated; without integration, life is not possible.”[…] “What we are concerned with is not the search for a liaison between brain as a physical ‘thing’ and some other entity, which we call ‘mind.’ We need to understand brain as a functioning tissue that generates feelings, moods, and self-awareness, that constitutes the basic ingredients of mind.”

Since self-process is both convergent and divergent, it can function both as the source and the target of its behavior and mentation. It is ever renewing since it is continuously generated as a result of spontaneous activation of cerebral cortex by the brainstem-diencephalic arousal system. The brainstem-cortical arousal system is crucial for the manifestation of a living, thinking self. Without such neurophysiological arousal, consciousness, sense of self, attention, and volition are not possible.

The self is a dynamic, composite, and self-renewing process. Because of its dynamic nature, self is continuously changing and adapting to its situational environment. The homeostatic and adaptive control of its arousal and behavior is influenced by the sleep-wake cycle[13]. Self is a composite of many modalities, with concurrent, neurodevelopmental streams, such as (a) a consumer by breathing, swallowing, and sexuality; (b) a mover-speaker-communicator by locomotion, gestures, and speech; (c) a sensor-perceiver-observer by all sensory modalities; (d) an image maker, a thinker by cognition, internal speech, imagination; (e) an organizer-coordinator of personal, peripersonal, and extrapersonal space-time and experience; (f) a recaller and manipulator of working memories and information; (g) a simulator-imitator and modeler of perceived, imagined, and projected experiences; and (h) an adapter to ever new environment and to itself in its current existence.

The current experience of an organism defines its present self, and the present self, in turn, shapes its current experience. Such a circular, multistream self is usually engaged in a relationship with someone or something, and is always embedded in a larger ecosystemic process. Such interactive, experiential space or life-space is represented in the brain and can be understood as personal (body and its activities), peripersonal (manually reachable space), and extrapersonal space beyond reach that can be imagined and projected[14]. Life-space is defined as the totality of possible events for one person at a particular time, which is a person’s possible options, together with the environment that contains them. It is like multiple streams being coordinated into a single, transient, convergent-divergent stream with its complex, coordination dynamics. This is why we have to understand self in terms of new biophysics, synergetics, and coordination dynamics for complex, natural systems.

The self or existential being can be behaviorally-mentally oriented in three complimentary modes of existence, namely (a) an egocentric, object-avoidant mode; (b) an allocentric, object-approach-and-explore mode; and (c) an ecosystemic, life-space or field mode, which is self-existent, globally oriented, and holistic. The latter holistic or existential mode can be self-actualized with a conflict-free sphere of ego or "I" that remains ever-the-same throughout an individual's conscious life.

Pfaff[15] recently proposed a brain arousal and information theory. He described a model with an acronym "BBURP," which stands for a Bilaterally symmetric, Bipolar (Bidirectional, ascending and descending) Universal (among vertebrates), Response Potentiating system. "Among all vertebrates, this system readies the animal or the human to respond to stimuli of all modalities, to initiate voluntary locomotion, and to react with feeling to emotional challenges...The totality of fundamental arousal is a function of generalized arousal plus an increasing function of specific arousal states (sex, hunger, thirst, pain, fear and so on)." He considers arousal as the most fundamental force of the nervous system and proposes an operational definition of arousal: "'Generalized arousal' is higher in an animal or human being who is: more alert to stimuli of all sorts, and more motorically active, and more reactive emotionally." Six neurotransmitter systems, using norepinephrine, dopamine, serotonin, acetylcholine, histamine, and orexin, work together to mediate brain arousal. These independent, but coordinated, arousal systems originate in the brainstem-limbic regions and converge in the nonspecific thalamus with dorsal and ventral paths to the associative and sensorimotor cortices.

Seeley and Sturm[16] described the ontogeny of self-representation: "From birth, humans iteratively construct the self, beginning with simple body state 'building materials' and layering on new skills until we learn to think abstractly about our selves, others, and the complex relations between them." The minimal self represents data available only to its owner including the current body state, and the conscious stream of internal dialogue and imagery. It is rooted in the ancient neural systems of the brain stem reticular formation and the limbic system. The authors describe three processing streams of the minimal self: (a) interoception maps the body states into consciousness, probably mediated by a neural network connecting insula, amygdala, anterior cingulate cortex, and the orbitofrontal cortex; (b) exteroception maps the external reality relying on bilateral, fronto-cingulo-parietal, spatial inputs, however, the attentional matrix is processed predominantly in the right parietal cortex; and (c) phrenoception or representations of spontaneous mental thoughts and acts are probably mediated by a default mode network. Such undirected, spontaneous brain network includes dorso- and ventromedial prefrontal cortex, paracingulate region, posterior cingulate/precuneus, lateral posterior parietal cortex, and the hippocampi. "If the minimal self describes self-representational acts that occur in the moment, the longitudinal self relates to elements of our extended individual life histories…The dorsomedial prefrontal cortex, which somehow participates in both semantic and episodic self-related memory, may be a node that helps bind the minimal and longitudinal selves together, marking elements of each as originating from or relating to the self."

Embodiment, the sense of being localized within one's physical body, is a fundamental aspect of the self. The phenomenon of embodied self is processed in distinct cortical areas viz. temporoparietal junction and the extrastriate body area[17]. The neural network that processes embodiment of self, its parts, and corporeal awareness include not only the posterior parietal-temporal cortex, but also premotor and motor cortices. Carruthers[18] proposes a model of the synchronous self, which includes the sense of control over one's body and mind, of being bounded in body and mind, of having perspective from within one's body and mind, and of being extended in time. Cavanna and Trimble[19] reviewed the role of the precuneus in self-processing. They suggest that the precuneus appears to play a crucial role in self-processing tasks, possibly through the involvement of mental imagery and the retrieval of self-related, episodic memories.

Damasio[20] proposed his/her solution to the conscious-mind problem with a movie metaphor: "The sense of self in the act of knowing emerges *within* the movie. Self-awareness is actually part of the movie and thus creates, within the same frame, the 'seen' and the 'seer', the 'thought' and the 'thinker'. There is no separate spectator for the movie-in-the-brain. The idea of spectator is constructed within the movie, and no ghostly homunculus haunts the theater. Objective brain processes knit the subjectivity of the conscious mind out of the cloth of sensory mapping. And because the most fundamental sensory mapping pertains to body states and is imaged as feelings, the sense of self in the act of knowing emerges as a special kind of feeling – the feeling of what happens in an organism caught in the act of interacting with an object."

The neural correlates of mental self were studied during yoga nidra relaxation meditation[21]. There was a relative decrease in blood flow in bilateral orbital and dorsolateral prefrontal, anterior cingulate, temporal, inferior parietal cortices, caudate nucleus, thalamus, pons, and cerebellar vermis, which are the structures associated with executive functions and working memory. Generally speaking, meditation shifted cortical activity from prefrontal to posterior regions. It was suggested that not only medial anterior, but also medial posterior cortical regions were essential for subjectivity. These regions seem to process a stable unified perspective of the organism relative to the environment, i.e., the "self".

Gazzaniga[22] suggested that a sense of self is constructed by the left hemisphere "*interpreter*" on the basis of input from a bihemispheric neural network. Zhu[23] concluded that human volition has at least three distinct aspects to its meaning as a mental act: (a) decision making, (b) action initiation, and (c) executive control. These are complexly evolving, multistream, dynamic processes. Volition, of course, has a lot to do with our intentionality and sense of conscious self. Zimmer[24] described a "self network" located in the medial prefrontal cortex, which draws together perceptions and memories of self and combines them into an ongoing feeling of being oneself. Precuneus is involved in retrieving autobiographical memories and the anterior insula becomes active when people look at pictures of their own faces.

In his recent book, *The Primacy of the Subjective*, Georgalis[25] proposed a new, nonphenomenal concept of minimal content, which is required to understand mind and language. He argues, "an agent constitutes minimal content and that this results in a unique intentional state: the '*Fundamental Intentional State'*." He thinks that a strict third-person methodology is untenable; it must be supplemented with a first-person, subjective methodology. Another landmark publication on *Subjectivity and Selfhood* is by Zahavi[26]. He stated, "According to Sartre, consciousness has two different modes of existence, a pre-reflective and reflective. Whereas the former is an immersed non-objectifying self-acquaintance, the latter is a detached objectifying self-awareness that introduces a phenomenological distinction between the observer and the observed. The former has priority since it can prevail independently of the latter, whereas reflective self-consciousness always presupposes pre-reflective self-consciousness." While describing Husserl's concept of subjectivity, he quotes: "To be a subject is to be in the mode of being aware of oneself.[…] An absolute existent is existent in the form of an intentional life – which, no matter what else it may be intrinsically conscious of, is at the same time, consciousness itself.[…] For the latter[the life of consciousness] not only a lived experiencing, continually streaming along; at the same time, as it streams along it is also immediately the consciousness of this streaming."

Zahavi addressed the relationship between consciousness and self. "An egological theory would claim that when I watch a movie, there is an object of experience (the movie), there is an experience (the watching), and there is a subject of experience, myself… In contrast, a non-egological theory would argue that experiences are egoless; they are anonymous mental events that simply occur, and minimal self-awareness should be understood as the acquaintance that consciousness has with itself and not as awareness of an experiencing self." The nonegological perspective is consistent with the giveness of a lived, prereflective conscious being, proprium, or ipseity. It is only when we adopt a distancing and objectifying attitude toward the experience, i.e., when we reflect on it, that an ego appears. The act of reflection is similar to the act of directed attention. It is the attentional vector that creates the observer-observed duality.

Finally, there is a recent philosophical proposal of the "*ultimate ground*" of experience and existence. So called "*physical*" and "*mental*" are two aspects of the same unitive reality. Pred[27] alluded to it as "*Onflow: organic monism of pure experience.*" "The ultimate represents the underlying, energetic, creative ground for process, for concrescence, and for the ongoing flow or advance of events. The ground does not exist apart from its activity and has three basic inseparable aspects: creativity, diversity and unity." Similar holistic perspectives of unitive, existential experience have been expressed[28,29,30,31,32]. Huxley described: "the divine ground of all existence is a spiritual absolute, ineffable in terms of discursive thought, but susceptible of being directly experienced and realized by the human being." One of the most eloquent expressions of such an advanced, integrated, and self-realized experience is given by Shankaracharya*:* "I am verily the ultimate existence, the source-energy of being, which is equanimous, tranquil, silent, self-existent, self-aware, joyously well, unchanging, unformed, undecaying, healthy, real, beyond-appearances, non-vacillating, pervasive, unspecified, restful, enduring, ever-free, stable, pure, quiescent, unlimited, and fearless of aging and death. I am not a mere representation of a body. This is the true insight into self"[33].

## MULTISTREAM NEUROBEHAVIORAL DEVELOPMENT OF SELF

The concept of a neurodevelopmental stream is now well established in neuroembryology and pediatric neuroscience. For instance, in the Denver II assessment scale for neurodevelopment of a child, from the newborn to 6 years of age, four major streams of growth are described: (a) gross motor, (b) fine motor adaptive, (c) language, and (d) personal-social. At each specific age, a child's growth can be assessed for normality according to these four neurodevelopmental streams. These streams evolve concurrently, from moment to moment, into a composite and dynamic, individual self, specific for that particular person and his/her developmental stage. The neurobehavioral development and the ontogeny of self are briefly described in a chronological order[34,35,36,37,38,39,40,41,42,43,44,45,46,47].

### Embryonic Behavioral Development

1. 4$^{th}$ week: The human heart begins to beat at 65 beats/min.
2. 4–12 weeks: The circadian clock in suprachiasmatic nucleus begins to oscillate.
3. 6$^{th}$ week: The superficial musculature is developed. Cutaneous innervation and sensitivity begins around the mouth, extending to nose, and chin, eyelids, palms of hands, genitalia, and soles of feet.
4. 7$^{th}$ week: The cortical synapses begin, which continue to grow rapidly until 5 years, slowly until 18 years, and then continue to grow periodically throughout life.

### Fetal Behavioral Development

1. 7–10 weeks: First muscle contractions, followed by lateral flexion movements of neck and trunk. Trigeminal nerve terminals are reactive to tactile stimuli. The face is recognizably human. Local stimuli may evoke squinting, opening of the mouth, incomplete finger closure, and flexion of toes. Swallowing begins.
2. 11–15 weeks: Fetal nose is well shaped and amniotic fluid circulates in the upper airways. Main olfactory system has mature neurons. Breathing motions appear and tactile stimulation elicits graceful movements.
3. 17–40 week: Appearance of grasp reflex. Fetal breathing movements induce displacement of fluids in the nasal airways so that the amniotic fluid in contact with chemoreceptors is continually renewed, marking the beginning of chemosensorium.
4. 24–30 weeks: Ability to suck develops. Eye opening occurs with sensitivity to light, but perception of form and color is not complete until long after birth.
5. 3–6 months: Spinal cord and brain stem reflexes are present. The fetus begins to swallow and absorb amniotic fluid.

6. 20–28 week: Fetus develops rest-activity cycles. Spontaneous and adaptive responses follow extensor-avoidance and flexor-approach behaviors. Neurosensory development with initially random, and then gradually synchronous, firing of retinal ganglion cells, auditory system, olfactory system, sensory-motor neurons in the spinal cord, brainstem and thalamus, associated with REM sleep in the third trimester. Gradually, sleep differentiates into REM and NREM type.
7. 24–36 weeks: Neural systems, including reticulothalamic, somatosensory (pain, touch), olfactory, auditory, limbic (comfort-emotion), and hippocampal (learning and memory), are functional. The auditory system is ready to learn and respond to meaningful sounds (language and music) by body movements and elevation of heart rate. These responses decrease after several repetitions, which is a sign of learning by habituation.
8. Before birth: From late gestation, the fetus has an ability to detect, selectively encode, memorize, and retrieve the odor information available in the womb.
9. 38 weeks prenatal to 6 months postnatal: Development of directional columns of primary visual cortex. These include columns for lines, patterns, movement, and direction. Visual system is functional and ready for visual experiences.

## Neonate-Child-Adult Behavioral Development

1. Moment of birth: With the loss of placental connection with mother, there is an urgent need to begin breathing. This happens within seconds after a normal delivery and achieves a normal respiratory rhythm within 1 min after birth.
2. Newborn at birth: Alert and ready to respond and nurse. Neonates are nearsighted and have a fixed focal length of about 8–12 in. Visual preferences for a human face and preferential turning to a female voice. First social gazing lasts for about 40 min.
3. 0.5–8 h after birth: Ability to regulate arousal and organization of six discrete behavioral states — quiet sleep, active sleep, drowsy, alert, fussy, and crying. Term-born infants show hedonic differentiation of odorants by displaying facial action indicative of acceptance or rejection.
4. Birth to 1 month: Sleep-wake cycles of about 90-min duration are distributed through 24 h of day-night periods. Eyes follow to midline. Visual contact with faces and objects. Turns toward a novel sound. Can encode, store, and retrieve information about events experienced.
5. 2 months: Infants are more awake during the day. Responsive smile. Track with eyes past midline. Organize experiences by primitive reflexes like rooting and grasping. Actions relate to immediate environment experienced by the sensory-motor system. When overstimulated, infants calm themselves by looking away, yawning, or sucking on their lips or hands.
6. 3–6 months: More awake during the day and more sleep at night. Coos and chuckles. Hand-mouth movements. Shows discrimination by interacting differently with each parent and strangers. Anticipates feeding. Begins to develop a mental model of how the world works.
7. 4 months: Reaches for objects. Tracks with eyes 180° horizontal and some vertical tracking. Rolls prone to supine. Bats at objects. Plays midline. Laughs. Orients to voice. Enjoys looking around. Associates experiences and related memories (sound of mother’s voice with her face).
8. 5–6 months: Rolls supine to prone. Sits supported. Unilateral reach. Raking with all fingers. Babbles in one consonant syllables. Attached to primary caregiver. Follows object trajectory and its memory. Begin to control part of their world and realize their authorship.
9. 7–8 months: Transfers objects from one hand to the other. Imitates sounds. Anticipates object trajectory. Finger feeds. Crawls by dragging pelvis on floor. “Dada” nonspecific. Development of object permanence (peek-a-boo games) and primary caregiver permanence and stranger anxiety.
10. 9–12 months: Develops “joint attention”, the ability to use gaze and gesture cues to share experiences with others, which matures by 18 months. Infants use these skills to develop theories of mind and to use others’ affective cues to guide their behavioral choices. Waves “bye bye”. Understands “no”. Uncovers hidden objects. Picks up small objects with pincer movement.

"Dada" and "Mama" specifically. Wernicke's temporal area for language comprehension myelinates before Broca's frontal area for language production. Primitive forms of explicit memory are thought to emerge late in the first year.

11. 11–12 months: Points with index finger. One-word, one-step command with gestures. Two words. Immature jargoning. Imitates. Develops reciprocal communication of asking and showing. Comes when called. Cooperates with dressing. Uses a cup.
12. 15–24 months: Runs, pivots, walks backwards. Scribbles. Removes shoes. Uses four to six words. Follows one-step command without gesture. Develops ability to pass mirror test and video self-recognition test. Piaget's sensorimotor development[48]. Erikson's basic trust vs. mistrust[49].
13. 18 months to 3 years: Walks upstairs with rail. Throws a ball. Imitates stroke on paper. Uses spoon with solids with little spilling. Takes off most clothes. Uses ten or more words. Combines two words. Understands two-part command. Names pictures. Points to body parts. Realizes that they are truly separate from their parents and therefore vulnerable. Toddlers begin to use personal pronouns (e.g., "I", "me", "you") in reference to self and others. They come to realize that people are psychological beings with private, internal worlds; they distinguish behavior from thinking, desire from action, and reality from pretense. Memory systems develop including episodic and autobiographical memory, which provide the foundation for a longitudinal representation of self. In order to relieve stress, they develop self-consoling habits like thumb sucking, rocking, head banging, and using transitional objects ("lovies"). Preoperational symbolic-representational stage, (Piaget). Autonomy vs. shame (Erikson).
14. 24 months: Uses 50 words. Names body parts. Kicks ball. Walks downstairs. Imitates vertical line. Puts on some clothes. Vocabulary of 50–300 words. 25% of speech understandable. Follows commands with two actions on two objects. Increased locomotion and expansion of vocabulary and learning to change their environment and people through gestures and words. Practice independence by walking away. Demanding what they want. Doing anything without realizing consequences. Develop temper tantrums.
15. 2–4 years: Draws circle with perseverating lines. Can tricycle. Dresses with supervision. Vocabulary of 900 words. Knows first and last name, age, sex, colors. Three- to four-word sentences. Asks "why" incessantly. 75% understandable speech. Understands cold, tired, and hungry. Rapid language development provides concrete feedback and self-relevant information that enhances self-concept. By 2 years, begins to use mental state words, and by 3 years, routinely speaks of thoughts, beliefs, and feelings. Three-year olds know that thinking occurs internally and cannot be seen. By age 4, they reliably pass the tests of "theory of mind".
16. 3–6 years: Initiative and enjoyment of new locomotor and mental powers. Problem solving. "Magical" thinking. Self-centered with concrete, visual thinking. Lack logical explanation of events. They think they cause events. By 5 years, can print their first name. Can understand and verbalize the concepts of size, shape, and numbers. Combine sentences with conjunction. Follow three simple instructions. Read few letters and can tell the meaning of familiar words. Learn about the social world through interactive play with siblings and peers. Develop "pretend" play, which can ease anxiety. Act as superheroes to defeat evil villains. Perceive death as reversible. By 3 years, start becoming less "self-focused" and more social, being aware of other's needs. By 4 years, they become protective of younger children. By 5 years, they may show some leadership. Preoperational intuition (Piaget). Initiative vs. guilt (Erikson).
17. 6–12 years: Drive to be successful academically and socially. School-aged children enter "concrete operational stage" of thinking, meaning they can only operate on things present or experienced. Their representations are more connected and logical. They understand classification, seriation, and conservation. Seeing relationships and hierarchies. Develop symbol-associative learning, rule-governed play, and increased awareness of social expectations. Learn complex cognitive skills leading to their self-esteem. Learn to delay gratification by impulse control. Learn to control and sustain attention during 1st and 2nd grades. A 6-year old can focus attention on a task for 15 min. A 9-year old should be able to pay attention for an hour. A 6-year old should be able to express feelings and thoughts. At 6 years, they learn to read and start understanding syntax and grammar. By 7 years, they can articulate all sounds of the language they are exposed to. Between 7 and 10 years, they learn irregular verbs, tenses, and expressive language. In the early grades, friendships are of the same sex, but as puberty approaches, friendships become heterosexual. Moral development and empathy continue to progress from the concrete level of rule following toward more independent thinking of right and wrong. As children learn to negotiate the rules of society, they may engage in behaviors of lying, cheating, and stealing to fulfill fantasies, save face, test limits, or fit into a peer group. Concrete, operational (Piaget). Industry vs. inferiority (Erikson).

18. 12–17 years: Establish self-identity and sexual orientation. Capacity for abstract thought, hypothetic-deductive reasoning. Can hypothesize about "what is possible" and develop strategies to solve problems. Increased ability to self-monitor and communicate thoughts and feelings. The adolescent develops an "*armor* of middle adolescence: the *helmet* of omniscience, the *breast-plate* of omni-potence and the *shield* of invincibility." Formal operational, hypothetic-deductive logic (Piaget). Identity vs. role confusion. (Erickson).
19. 17–30 years: Intimacy vs. isolation (Erikson).
20. 30–60 years: Generativity vs. stagnation (Erikson).
21. After 60 years: Ego integration vs. despair (Erikson).

## TRIMODAL ORIENTATION OF THE MULTISTREAM SELF

The self process can be oriented in three complimentary modes of being-in-existence, namely (a) egocentric, object-avoidant mode; (b) allocentric, object-approach-and-explore mode; and (c) an ecosystemic, noninteractive, globally oriented, conflict-free, existential mode. The first two interactive modes of reflective consciousness create the subject-object duality, whereas, the noninteractive, prereflective, consciousness is restful, spontaneous, nondual, and existential. Such a mode can be intuitive and creative. The existential, conflict-free sphere of self-awareness remains the same throughout an individual's conscious life.

The ego or "I" is viewed as the conscious sense of self. According to the "Ego psychology" in psychoanalysis, the main function of ego is to control impulses and deal with the situational reality. Ego also contains a "conflict-free sphere" of functioning and has its own store of energy with which to pursue goals that are independent of instinctual wishes. This is in contrast to "Id psychology", which focuses only on the primitive instincts of sex and hostility.

Kraft[50] described the process of creative revelations: "A little relaxation and distance change the mind's perspective on the problem – without us being aware of it. This change of perspective allows for alternative insights and creates the preconditions for a fresh, and perhaps more creative, approach. The respite seems to allow the brain to clear away thought barriers by itself. At some point, newly combined associations break into consciousness, and we experience sudden, intuitive enlightenment." Such exceptional creative-intuitive moments have been described in meditative and artistic states[31,32,33].

Finally, Monnier has well summarized the important concepts of psychoneurobiology: He proposes "a scientific concept of the *indissociability of the psyche* (mind + affectivity) *and its brain* (neuronal and neurochemical systems). This indissociability is revealed: (1) by the concurrent construction of the psyche and the brain during ontogenic development; (2) by the synergism of the psychic and cerebral activities; (3) by the isomorphism of mental/affective and cerebral/humoral structures; and (4) by the impossibility of detecting with scientific methods mental/affective and cerebral/humoral manifestations after death"[51].
